# Supplementary material for: Opposing roles of pseudokinases NRBP1 and NRBP2 in regulating L1 retrotransposition
Source: Nat Commun. 2025 Jul 11;16:6327. doi: 10.1038/s41467-025-61626-z (PMC12254500; doi:10.1038/s41467-025-61626-z)
Supplement: Supplementary file 1 — Supplementary Information [file 41467_2025_61626_MOESM1_ESM.pdf]

Supplementary information for

Opposing roles of pseudokinases NRBP1 and NRBP2 in regulating L1 retrotransposition

Wei Yang[1,2*], Shaobo Cong[1*], Ruoyao Li[1], Jennifer Schwarz[3,4], Thilo Schulze[5], Raban A. Gevelhoff[1,6], Xinyan Chen[1,6], Sara Ullrich[1], Kristina Falkenstein[1], Denis Ott[1], Pia Eixmann[1], Angelica Trentino[1], Antje Thien[1], Thierry Heidmann[7], Ekkehard Schulze[1], Bettina Warscheid[3,8], Ralf Baumeister[1,6,9,10#] and Wenjing Qi[1#]

[1]Bioinformatics and Molecular Genetics, Institute of Biology III, Faculty of Biology, Albert-Ludwigs-University Freiburg, Germany

[2]College of Food Science and Engineering, Shandong Agricultural University, Shandong Engineering Research Center of Food Nutrition and Active Health, Taian 271018, People's Republic of China

[3]Biochemistry-Functional Proteomics, Institute of Biology II, Faculty of Biology, Albert-Ludwigs-University Freiburg, Germany

[4]European Molecular Biology Laboratory (EMBL), Heidelberg, Germany

[5]Department of Animal Evolution and Biodiversity, Georg-August-Universität Göttingen, Untere Karspüle 2, Göttingen 37073, Germany

[6]Spemann Graduate School of Biology and Medicine (SGBM), Albert-Ludwigs-University Freiburg, Freiburg, 79104, Germany

[7]CNRS UMR 9196, Laboratory of Molecular Physiology and Pathology of Endogenous and Infectious Retroviruses, Gustave Roussy, University Paris-Saclay, Villejuif, France

[8]Biochemistry II, Theodor-Boveri-Institute, Biocenter, University of Würzburg, 97074, Würzburg, Germany

[9]Signalling Research Centers BIOSS and CIBSS, Albert-Ludwigs-University Freiburg, Freiburg, 79104, Germany

[10]Center for Biochemistry and Molecular Cell Research, Faculty of Medicine, Albert-Ludwigs-University Freiburg, Germany

[*]These authors contributed equally.

[#]Correspondence: wenjing.qi@biologie.uni-freiburg.de, baumeister@celegans.de, Bio3/Bioinformatics and Molecular Genetics, University of Freiburg, Schänzlestrasse 1, D-79104 Freiburg, Germany

Supplementary information includes:

Supplementary Figs. 1 to 12

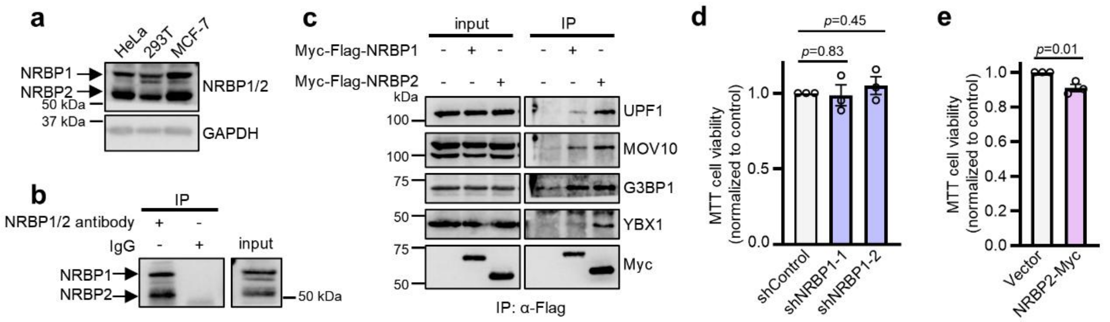

Supplementary Fig. 1 NRBP1 and NRBP2 interact with L1-encoded ORF1p and other ORF1p interactors.

a, Western blot analysis of endogenous NRBP1 and NRBP2 in HeLa, HEK293T and MCF-7 cells using an antibody recognizing both pseudokinases (NRBP1/2). GAPDH was used as a loading control. Shown is one representative Western blot of three independent experiments. Uncropped blots in Source Data.

b, Validation of the NRBP1/2 antibody used in mass spectrometry (MS) interactome analysis showing recognition of both NRBP1 and NRBP2. Representative blot is shown from three independent experiments. Uncropped blots in Source Data.

c, Both NRBP1 and NRBP2 are associated with multiple known L1 interactors or regulators. HEK293T cells were transfected with the indicated plasmids. Flag antibody was used to pull down Myc-Flag-NRBP1 or Myc-Flag-NRBP2. Co-precipitated endogenous proteins were detected by using respective antibodies. Myc-Flag-NRBP1 and Myc-Flag-NRBP2 were detected with Myc antibody. Shown is one representative data from three independent experiments. Uncropped blots in Source Data.

d, NRBP1 knockdown has no significant impact on cell viability. $n = 3$ biological replicates. Data are mean ± SEM. Two-sided unpaired t-test; no multiple comparison adjustment.

e, NRBP2 overexpression shows a modest decrease in cell viability. $n = 3$ biological replicates. Data are mean ± SEM. Two-sided unpaired t-test; no multiple comparison adjustment.

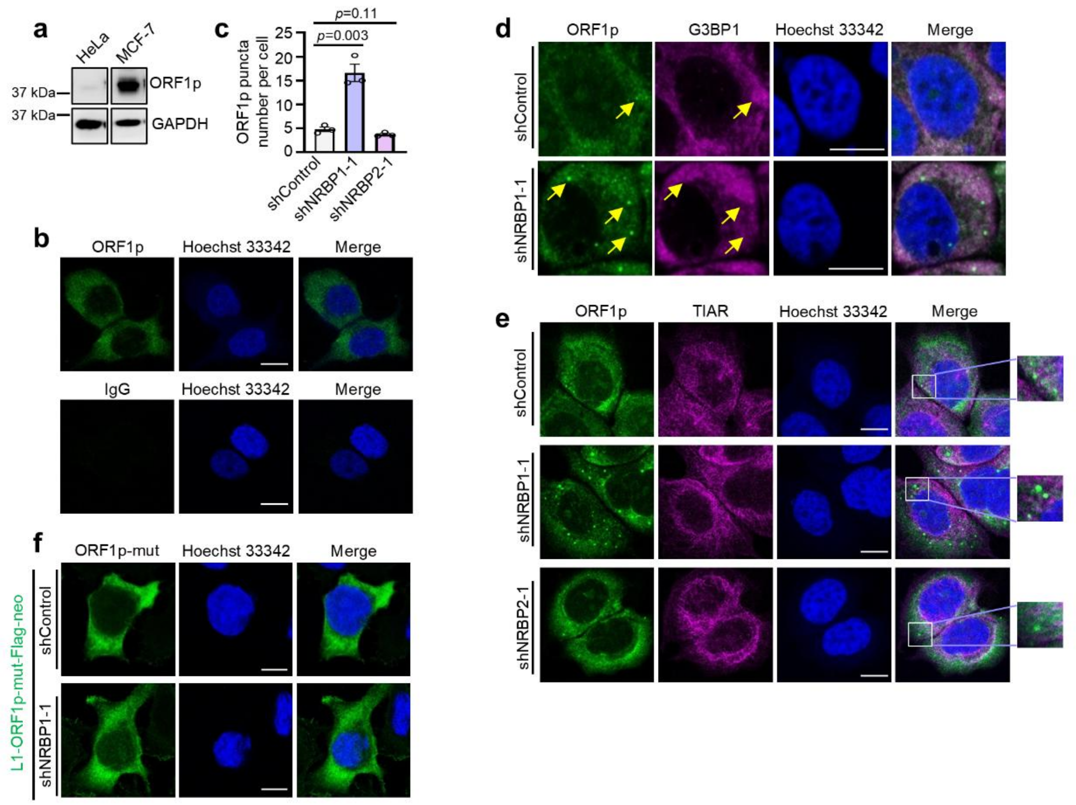

Supplementary Fig. 2 ORF1p puncta induced by NRBP1 knockdown do not colocalize with G3BP1 or TIAR.

a, MCF-7 cells show higher ORF1p level than HeLa cells. GAPDH served as a loading control. Shown is a representative blot from three independent experiments. Uncropped blots in Source Data.

b, The ORF1p antibody staining (green) shows a higher signal intensity than the IgG control in MCF-7 cells. Nuclei are stained blue. Scale bar 10 µm. Shown is one representative image of three independent experiments.

c, Quantification of ORF1p puncta per cell. The figure is related to main Fig. **2a**. Each dot represents an average result of one biological replicate (n = 3). The number of cells counted in each replicate is as follows: shControl (n = 13, 14, 11); shNRBP1-1 (n = 32, 8, 7); shNRBP2-1 (n = 30, 30, 7). Data are mean ± SEM. Two-sided unpaired t-test; no multiple comparison adjustment.

d, ORF1p foci induced by NRBP1 knockdown do not co-localize with G3BP1 in MCF-7 cells. The yellow arrows point to ORF1p foci in the green channel and the absence of G3BP1 enrichment in the magenta channel. Shown are representative immunofluorescence staining of endogenous ORF1p and G3BP1 proteins with their respective antibodies. Nuclei are stained blue. Scale bar 10 µm. n = 3 independent experiments.

e, ORF1p (green) foci induced by NRBP1 knockdown do not co-localize with TIAR (magenta) in MCF-7 cells. Nuclei are stained blue. Scale bar 10 µm. Shown are representative images of three independent experiments.

f, Knockdown of NRBP1 has no effect on localization of the transfected ORF1p (N157A/R159A)-mutant (green) that has lost its RNA-binding activity in HeLa cells. Experiment was repeated twice with similar results. Nuclei are stained blue. Scale bar 10 µm.

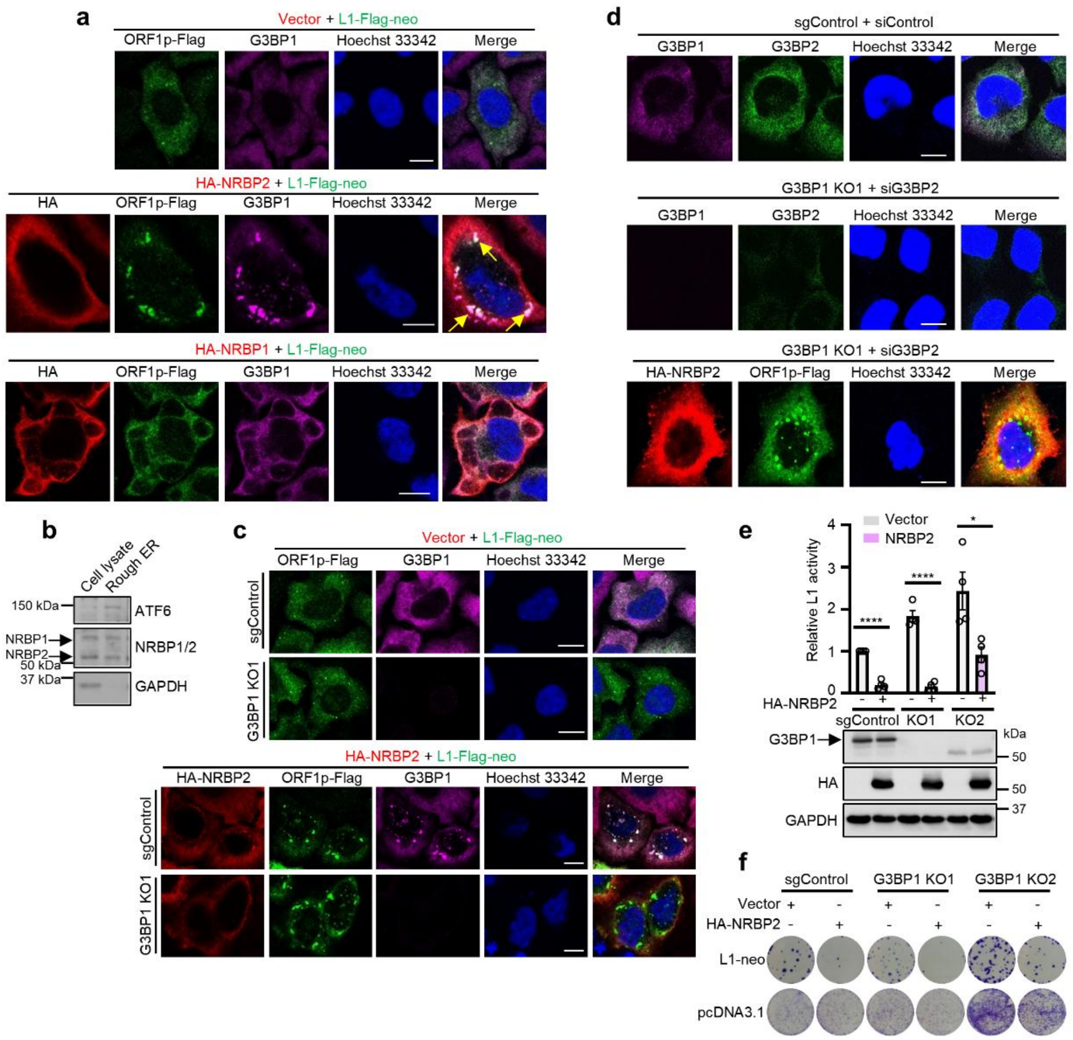

Supplementary Fig. 3 NRBP2-mediated L1 inhibition is independent of G3BP1.

a, HA-NRBP2 (red) overexpression results in an enrichment of ORF1p-Flag (green) in cytoplasmic puncta in HeLa cells. The yellow arrows point to colocalized ORF1p and G3BP1 (magenta) foci. Nuclei are stained blue. Scale bar 10 µm. Shown are representative images of three independent experiments.

b, Both NRBP1 and NRBP2 are present in the rough endoplasmic reticulum (ER) of HeLa cells. Rough ER fraction was isolated from HeLa cells. Western blot was performed to detect the presence of NRBP1 and NRBP2. Unprocessed ATF6, an established ER marker, was used to confirm the enrichment of the rough ER fraction.

Shown is one representative Western blot of three independent experiments. Uncropped blots in Source Data.

c, Induction of transfected ORF1p-Flag (green) enriched foci by NRBP2 (red) overexpression is independent of G3BP1 (magenta) in HeLa cells. $n = 3$ biological replicates with similar results. Nuclei are stained blue. Scale bar 10 µm.

d, NRBP2 overexpression induces transfected ORF1p-Flag foci formation in the absence of both G3BP1 and G3BP2 in HeLa cells. $n = 2$ biological replicates with similar results.

e, Inhibition of L1 retrotransposition by NRBP2 is independent of G3BP1. Quantification of the colony assay is shown in the top panel and one representative Western blot result is shown in the bottom panel. $n = 4$ biological replicates. Data are mean ± SEM. Two-sided unpaired t-test; no multiple comparison adjustment. $p = 3.69 \times 10^{-6}$ (sgControl), $p = 2.28 \times 10^{-5}$ (KO1), $p = 0.02$ (KO2). $*p < 0.05$, $****p < 0.0001$.

f, One representative picture of the colony assay depicted in **(e)**.

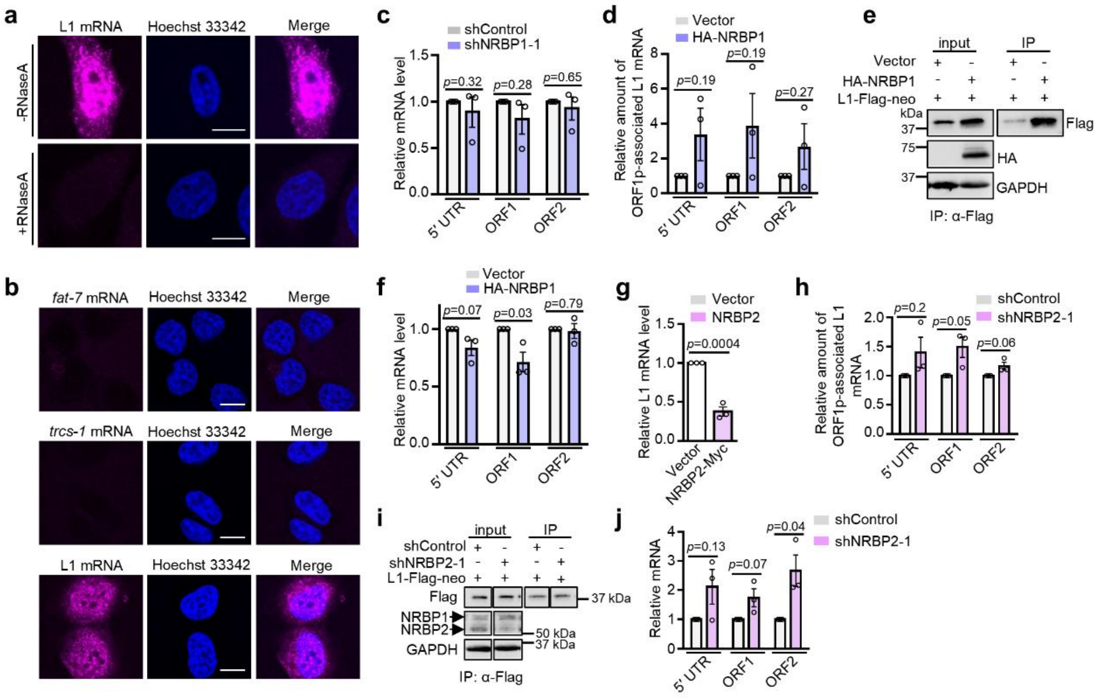

Supplementary Fig. 4 NRBP2 knockdown moderately enhanced L1 mRNA and ORF1p association.

a, RNase A treatment reduces L1 mRNA signal (magenta) detected by smFISH in MCF-7 cells. Shown are representative images of three independent experiments. Nuclei: blue. Scale bar 10 µm.

b, Probes detecting L1 mRNA (magenta) yield stronger signals than those targeting *C. elegans fat-7* or *trcs-1* mRNAs in MCF-7 cells. *n* = 2 biological replicates with similar results. Nuclei: blue. Scale bar 10 µm.

c, NRBP1 knockdown does not alter endogenous L1 mRNA levels (normalized to GAPDH) in MCF-7 cells. *n* = 3 biological replicates. Data are mean ± SEM. Two-sided unpaired t-test; no multiple comparison adjustment.

d, RIP-qPCR quantifying L1 mRNA bound to Flag-tagged ORF1p expressed from the L1-Flag-neo following NRBP1 overexpression in HeLa cells. *n* = 3 biological replicates. Data are mean ± SEM. Two-sided unpaired t-test; no multiple comparison adjustment.

e, Western blot confirms HA-NRBP1 transfection and ORF1p IP in **(d)**. Uncropped blots in Source Data.

f, Effect of NRBP1 overexpression on L1 mRNA levels. NRBP1 was co-transfected with L1-Flag-neo in HeLa cells. L1 mRNA levels were normalized to hygromycin-resistance gene encoded by the L1 reporter[1]. n = 3 biological replicates. Data are mean ± SEM. Two-sided unpaired t-test; no multiple comparison adjustment.

g, Overexpression of NRBP2 reduced L1 mRNA level. NRBP2 was co-transfected with L1-Flag-neo in HeLa cells. Transfected L1 mRNA levels were quantified by qRT-PCR using neo-specific primers and normalized to the hygromycin-resistance gene. n = 3 biological replicates. Data are mean ± SEM. Two-sided unpaired t-test; no multiple comparison adjustment.

h, NRBP2 knockdown slightly increases ORF1p and L1 mRNA association. L1-Flag-neo was transfected into NRBP2 knockdown HeLa cells. Co-precipitated mRNA was quantified by qRT-PCR. n = 3 biological replicates. Data are mean ± SEM. Two-sided unpaired t-test; no multiple comparison adjustment.

i, Western blot shows NRBP2 knockdown and ORF1p-Flag IP efficiency in **(h)**. Uncropped blots in Source Data.

j, NRBP2 knockdown moderately increases L1 mRNA levels. L1-Flag-neo was transfected into NRBP2 knockdown HeLa cells. L1 mRNA was normalized to the hygromycin-resistance gene. n = 3 biological replicates. Data are mean ± SEM. Two-sided unpaired t-test; no multiple comparison adjustment.

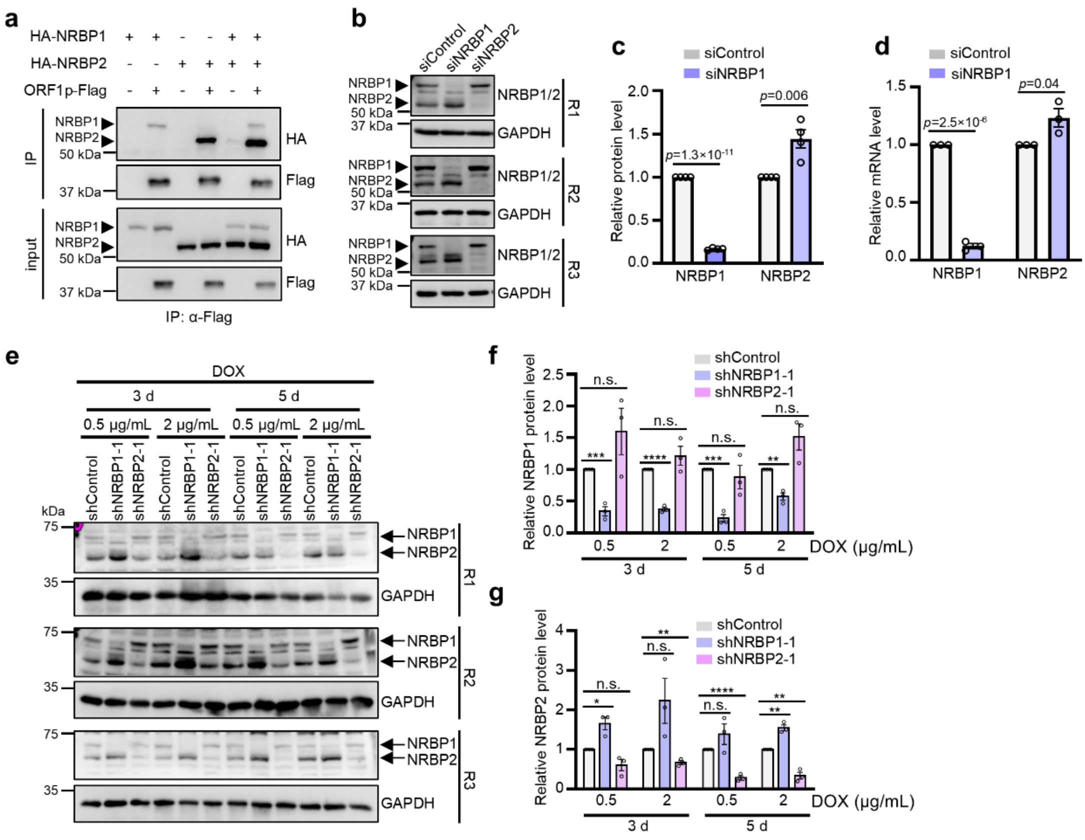

Supplementary Fig. 5 NRBP2 negatively regulates NRBP1 to inhibit L1 retrotransposition.

a, Overexpression of NRBP1 or NRBP2 does not interfere with binding of their ortholog counterpart to ORF1p. Co-IP was performed in HeLa cells by using Flag antibody. Shown is one representative result of three independent experiments. Uncropped blots in Source Data.

b, Additional three biological replicates showing that NRBP1 and NRBP2 negatively regulate each other's protein expression. One biological replicate is shown in main Fig. **3d**. R stands for Replicate. Uncropped blots in Source Data.

c, NRBP1 knockdown increases protein level of NRBP2. Shown is quantification of four replicates in **(b)** and main **Fig. 3d.** Data are mean ± SEM. Two-sided unpaired t-test; no multiple comparison adjustment.

d, NRBP1 knockdown increases NRBP2 mRNA levels (normalized to GAPDH) in HeLa cells. $n = 3$ biological replicates. Shown are mean ± SEM. Two-sided unpaired t-test; no multiple comparison adjustment.

e, Exploring mutual negative regulation between NRBP1 and NRBP2 by using shRNA in HeLa cells. The cells were treated with different doses of Doxycycline (DOX) for the indicated days before carrying out the Western blot. Results are from three biological replicates. R stands for Replicate. Uncropped blots in Source Data.

f, Quantification of relative NRBP1 protein levels shown in **(e)**. p values for comparisons between shControl and shNRBP1 (left to right): 0.0008, 2.9×10^{-5}, 0.0002, and 0.002; and between shControl and shNRBP2 (left to right): 0.18, 0.23, 0.55, and 0.07. n.s., not significant, $**p < 0.01$, $***p < 0.001$, $****p < 0.0001$. Shown are mean ± SEM. Two-sided unpaired t-test; no multiple comparison adjustment.

g, Quantification of relative NRBP2 protein levels shown in **(e)**. p values for comparisons between shControl and shNRBP1 (left to right): 0.01, 0.1, 0.2, and 0.002; and between shControl and shNRBP2 (left to right): 0.05, 0.003, 0.0002, and 0.002. n.s., not significant, $*p < 0.05$, $**p < 0.01$, $****p < 0.0001$. Shown are mean ± SEM. Two-sided unpaired t-test; no multiple comparison adjustment.

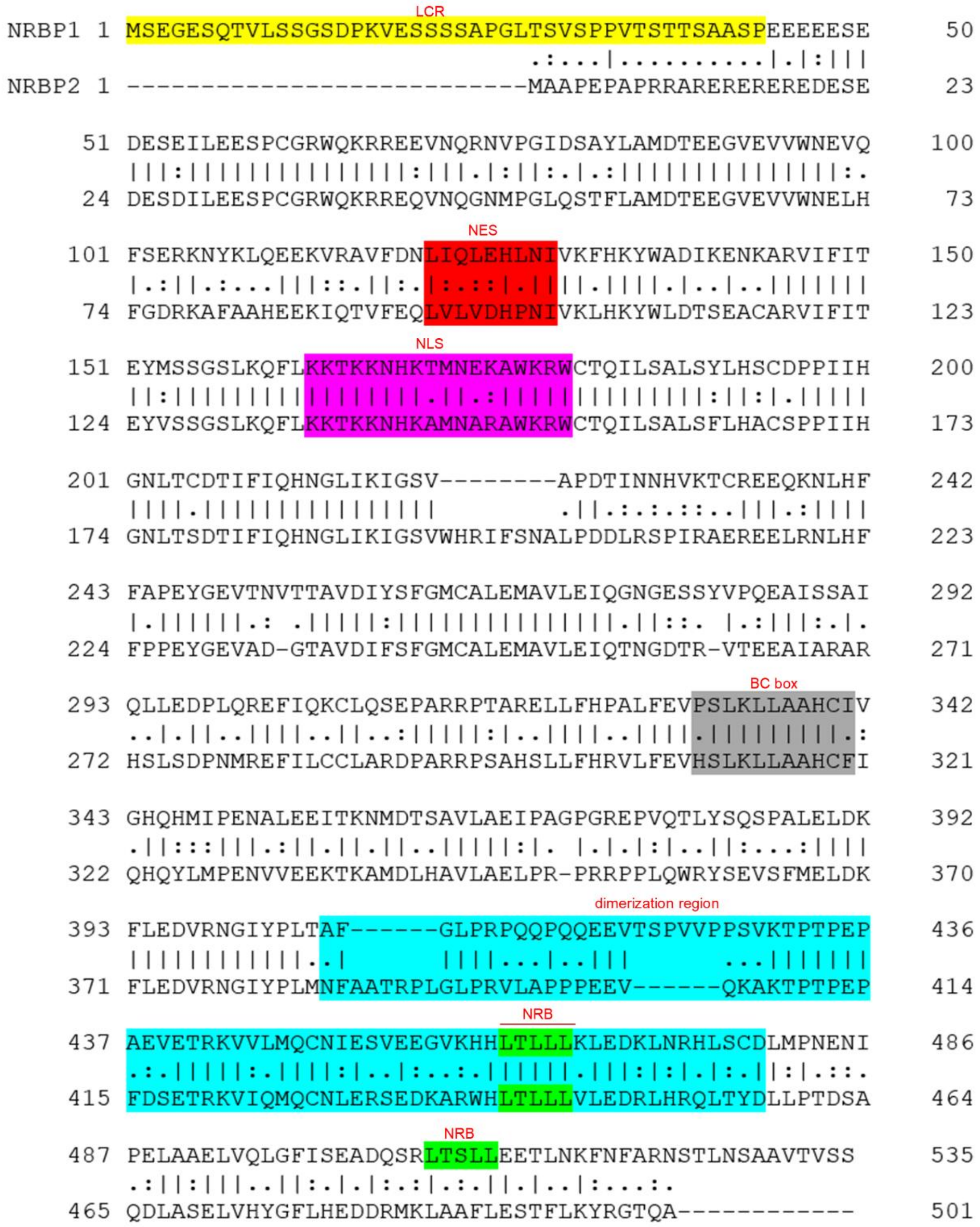

Supplementary Fig. 6 Alignment of the amino acid sequences of NRBP1 and NRBP2.

Yellow: LCR (low complexity region)[2-5]; red: NES (nuclear export signal); magenta: NLS (nuclear localization signal); gray: BC box (Elongin BC-binding motif); blue: dimerization region; green: NRB (nuclear receptor-binding motif)[6, 7].

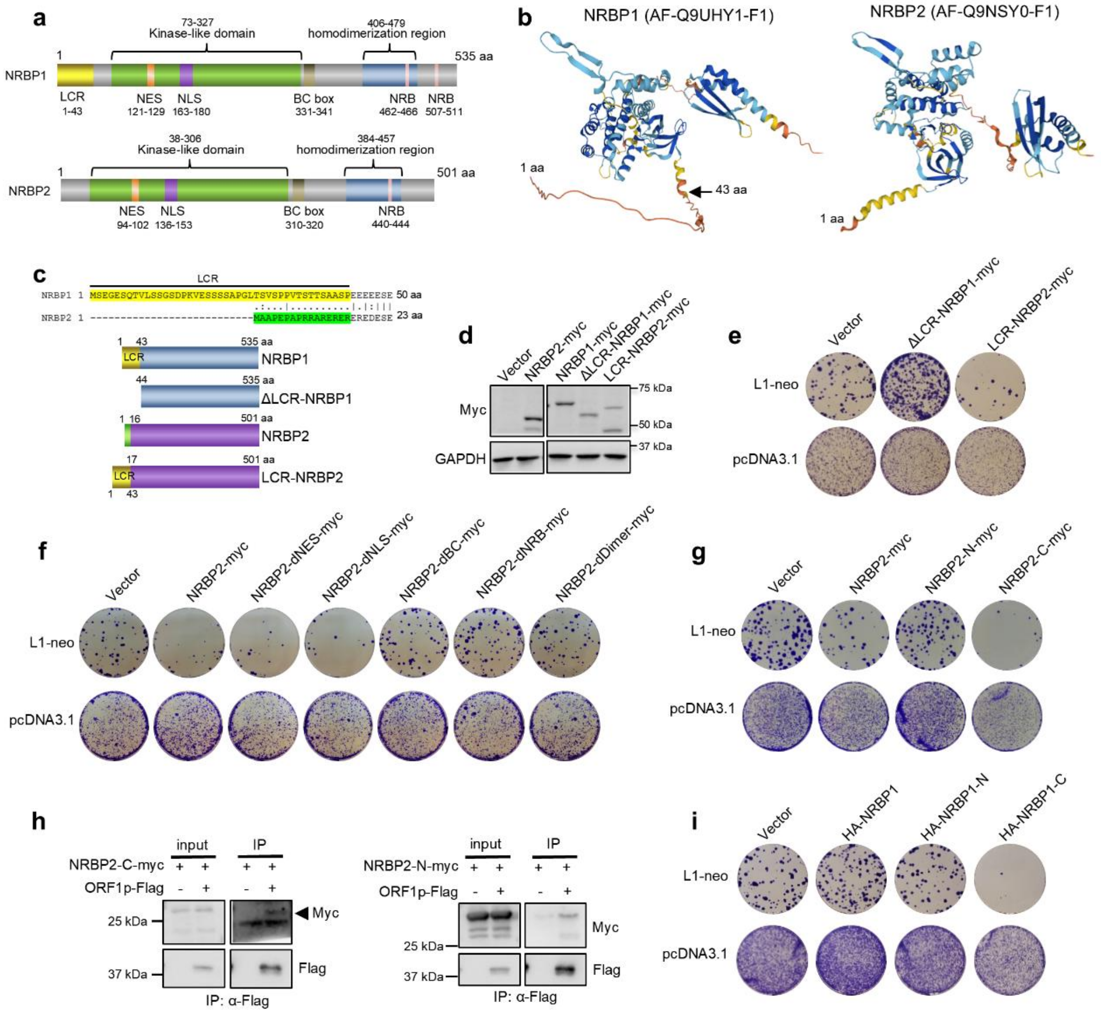

Supplementary Fig. 7 The C-terminal halves of both NRBP2 and NRBP1 negatively regulate L1 retrotransposition.

a, Diagram illustrations of domains / motifs in human NRBP1 and NRBP2 proteins. LCR: low complexity region; NES: nuclear export signal; NLS: nuclear localization signal; BC box: Elongin BC-binding motif; NRB: nuclear receptor-binding motif.

b, NRBP1 structure prediction by AlphaFold (AF-Q9UHY1-F1) reveals a potential unstructured N-terminal low complexity region (LCR).

c, A simple schematic diagram of NRBP1, NRBP2 and their mutants for the low complexity region (LCR)-swapping experiment.

d, A representative Western blot to show expression of NRBP1, NRBP2 and their mutants in **(c)** and **(e)**. Experiment was independently repeated twice with consistent results. Uncropped blots in Source Data.

e, Low complexity region (LCR)-swapping does not interfere with the regulatory roles of NRBP1 and NRBP2 in L1 mobility. Shown is one representative picture of the colony assay to examine L1 mobility. Experiment was independently repeated twice with consistent results.

f, NRB and dimerization region of NRBP2 are essential for its inhibitory role on L1. Shown is one representative picture of the colony assay depicted in main **Fig. 4b**. Experiment was independently repeated five times.

g, The C-terminal half of NRBP2 is necessary and sufficient to inhibit L1. Shown is one representative picture of the colony assay depicted in main **Fig. 4c**. The experiment was independently performed twice for NRBP2 and three times for NRBP2-N and NRBP2-C, with consistent results.

h, Both C- and N-terminal halves of NRBP2 interact with ORF1p in HEK293T cells. The experiment was independently performed twice with consistent results. Uncropped blots in Source Data.

i, The C-terminal half of NRBP1 functions oppositely to the full-length NRBP1 and inhibits L1. Shown is one representative picture of the colony assay depicted in main **Fig. 4e**. The experiment was independently performed six times for NRBP1, three times for NRBP1-N, and four times for NRBP1-C.

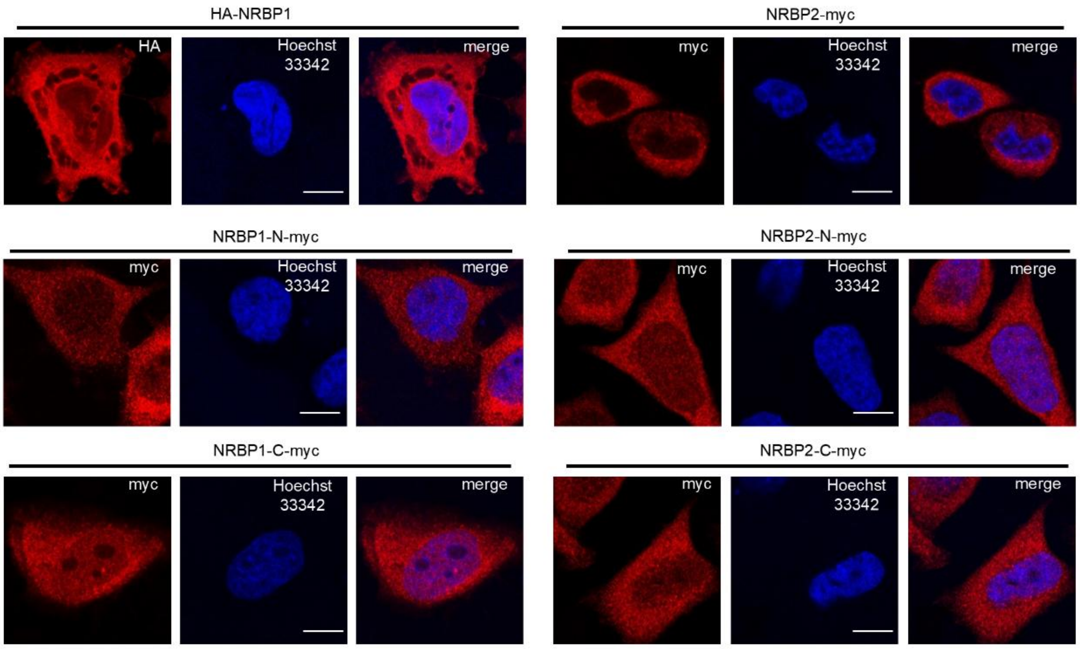

Supplementary Fig. 8 Subcellular localization of overexpressed HA-NRBP1, NRBP2-Myc and their respective N-terminal and C-terminal halves in HeLa cells. Immunofluorescence staining was performed with either HA or Myc antibody. Nuclei are stained blue. Scale bar 10 μm.

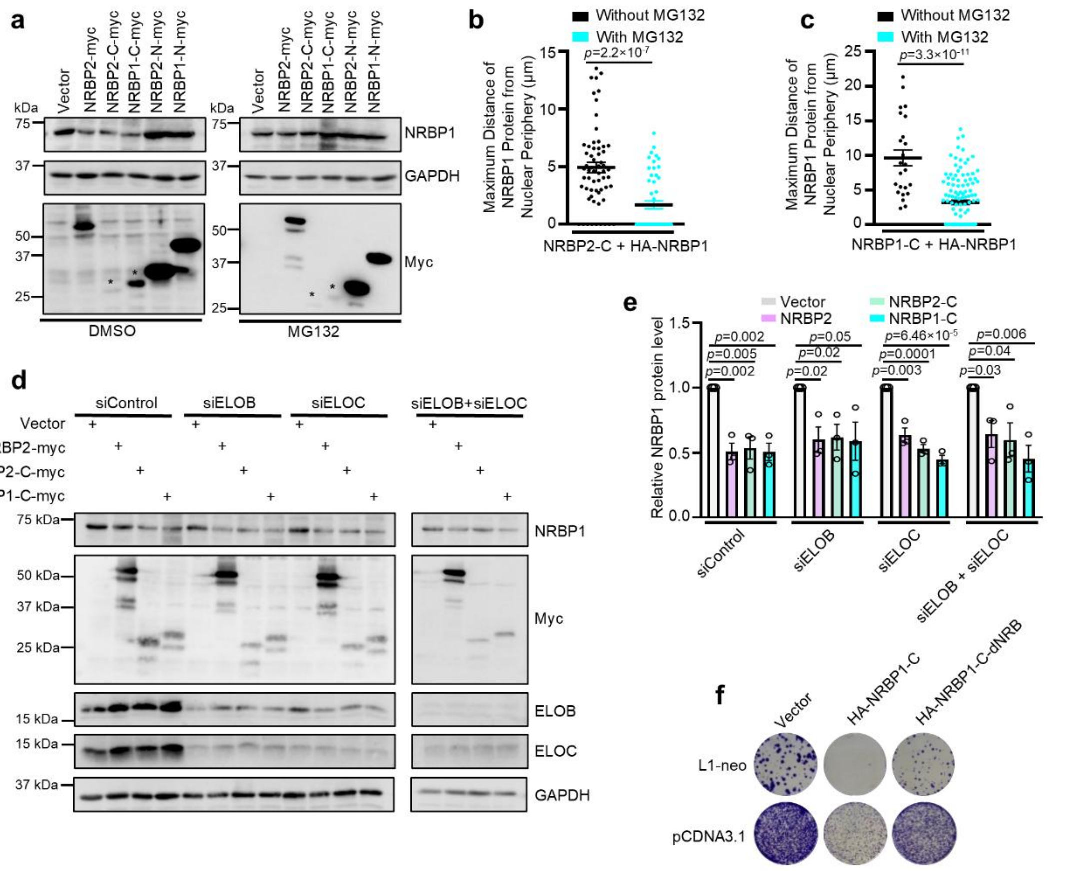

Supplementary Fig. 9 NRBP2 promotes NRBP1 degradation independently of the Elongin B/C E3 ubiquitin ligase complex.

a, Both the full-length NRBP2 and the C-terminal half of NRBP1 or NRBP2 reduce protein levels of full-length NRBP1 in HeLa cells and this could be blocked with MG132 treatment. Shown is one representative Western blot result used for the quantification in the main **Fig. 5d**. n = 3 biological replicates with consistent results. Uncropped blots in Source Data.

b, c, MG132 treatment restores perinuclear localization of NRBP1 displaced by either NRBP2-C **(b)** or NRBP1-C **(c)**. Quantification corresponds to the representative images in main **Fig. 5e**. Each dot represents one cell. NRBP1 redistribution was evaluated by measuring its maximal cytoplasmic extension from the nuclear envelope. Data are summarized from two biological replicates.

d, Both full-length NRBP2 and the C-terminal half of NRBP1 or NRBP2 promote NRBP1 decay independently of ELOB and ELOC. In line with prior findings, reducing either ELOB or ELOC resulted in a decrease in the protein levels of the other[8, 9]. Shown is one representative Western blot result used for the quantification in **(e)**. *n* = 3 biological replicates with consistent results. Uncropped blots in Source Data.

e, Quantification of three independent Western blot experiments from **(d)**. Data are mean ± SEM. Two-sided unpaired t-test; no multiple comparison adjustment.

f, The nuclear receptor-binding (NRB) motifs in the C-terminal half of NRBP1 are essential to inhibit L1. Shown is one representative picture of the colony assay depicted in the main **Fig. 5k**. *n* = 3 biological replicates with consistent results.

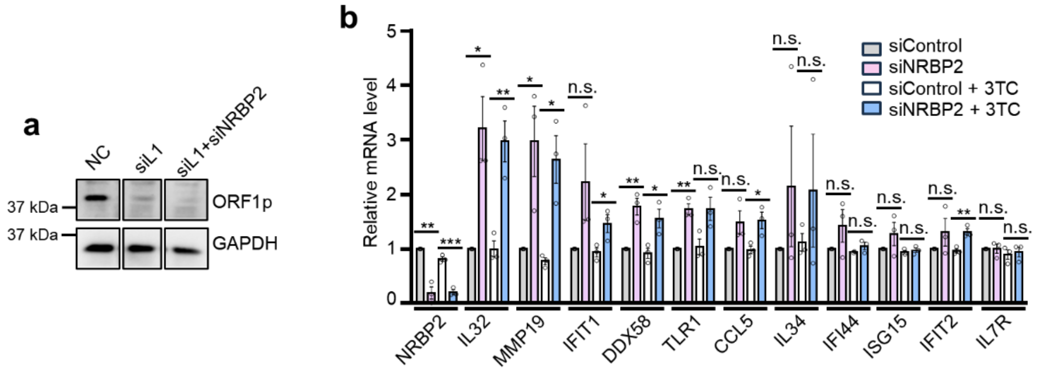

Supplementary Fig. 10 Down-regulation of NRBP2 activates inflammatory and type I interferon genes partially via L1 mRNA.

a, L1 siRNA efficiently reduces endogenous L1 expression level, as indicated by decreased ORF1p level in HeLa cells. $n = 2$ biological replicates with consistent results. Uncropped blots in Source Data.

b, Upregulation of immune-related genes by NRBP2 knockdown is not dependent on L1 cDNA. HeLa cells were transfected with siNRBP2 in the presence or absence of 3TC, a reverse transcriptase inhibitor that blocks L1 cDNA synthesis. qRT-PCR was used to quantify mRNA levels of the indicated genes, which were normalized to GAPDH. $n = 3$ biological replicates. Data are mean ± SEM. Two-sided unpaired t-test; no multiple comparison adjustment. n.s., not significant. * $p < 0.05$, * * $p < 0.01$, * * * $p < 0.001$. p values for siNRBP2 *vs.* siControl and siNRBP2 + 3TC *vs.* siControl + 3TC: NRBP2 (0.002, 0.0005), IL32 (0.02, 0.008), MMP19 (0.04, 0.01), IFIT1 (0.16, 0.04), DDX58 (0.007, 0.03), TLR1 (0.002, 0.053), CCL5 (0.09, 0.03), IL34 (0.36, 0.42), IFI44 (0.23, 0.21), ISG15 (0.27, 0.57), IFIT2 (0.29, 0.01), IL7R (0.98, 0.73).

```
NRBP1             1 M-----SEGESQT---VLSSGSDPKVESSSSAPGL----TSVSPPVTSTTSAAS------
NRBP2             1 M---------------------------------------AAPEPAPRRARERE------
C. elegans        1 M----VSSGEERT--------------------------AAGKTPIGDDAASDS------
D. melanogaster   1 M-----SNSQANA---GISGST-VADEPIQHHPSLAAGPVSASCPAATPPSQSTQQPPPH
A. queenslandica  1 MPRSPPSSQQTQAPPGGLSSSS--TTAASTGVPEL---PVATSEPVTGVSHEEKTKGP--

NRBP1            43 --------------------------------PEEEEESEDESEILEESPCGRWQKRREE
NRBP2            16 --------------------------------REREDESEDESDILEESPCGRWQKRREQ
C. elegans       25 -------------------------------------DADGAEEILEESPDKRWSKRREQ
D. melanogaster  52 IVSASTADAGSSAAVGVGVVAGSEGVNLDSSPRESGDDSEDESEILEESPCGRWLKRREE
A. queenslandica 54 --------------------------GADEVVEDEEEEEEDDEKILEIGHNGRWQKINHQ

NRBP1            71 VNQRNVPGIDSAYLAMDTEEGVEVVWNEVQFSERKNYKLQEEKVRAVFDNLIQLEHLNIV
NRBP2            44 VNQGNMPGLQSTFLAMDTEEGVEVVWNELHFGDRKAFAAHEEKIQTVFEQLVLVDHPNIV
C. elegans       48 VKQRDVPGIDVAYLAMDNETGNEVVWNEVQFSERKNFRAQEEKINAVFDNLTQLVHTNLV
D. melanogaster 112 VDQRDVPGIDCVHLAMDTEEGVEVVWNEVQYASLQELKSQEEKMRQVFDNLLQLDHQNIV
A. queenslandica 88 VT-RDVPGIDDAYLAMDTEEGVEV-------------------------------HQNIV

NRBP1           131 KFHKYWADIKE-NKARVIFITEYMSSGSLKQFLKKTK--KNHKTMNEKAWKRWCTQILSA
NRBP2           104 KLHKYWLDTSE-ACARVIFITEYVSSGSLKQFLKKTK--KNHKAMNARAWKRWCTQILSA
C. elegans      108 KFHKYWTDSKS-EKPRIIFITEYMSSGSMSAFLQRTR--KAGSSLSIKAWKKWTTQILSA
D. melanogaster 172 KFHRYWTDTQQAERPRVVFITEYMSSGSLKQFLKRTK--RNAKRLPLESWRRWCTQILSA
A. queenslandica 116 NFYDFWHDKVN-SKDRLVFITEYITSGSLAQFLKKNKRVKGTNSISDKIWRRWCRQILSA

NRBP1           188 LSYLHSCDPPIIHGNLTCDTIFIQHNGLIKIGSV--------APDTINNHVK----TCRE
NRBP2           161 LSFLHACSPPIIHGNLTSDTIFIQHNGLIKIGSVWHRIFSNALPDDLRSPIR----AERE
C. elegans      165 LNYLHSSDPPIIHGNLTCNTVFIQQNGLIKIGCV--------APDAINHHVK----TCRE
D. melanogaster 230 LSYLHSCSPPIIHGNLTCDSIFIQHNGLVKIGSV--------VPDAVHYSVRRGRERERE
A. queenslandica 175 LSYLHK-NE-IIHGNLSLASIFIQHNGLVKIGSV--------SPNAIHQHVK---TKNRE

NRBP1           236 EQKNLHFF-APEYGEVTN--VTTAVDIYSFGMCALEMAVLEIQ---GNGESS---YVPQE
NRBP2           217 ELRNLHFF-PPEYGEVAD---GTAVDIFSFGMCALEMAVLEIQ---TNGDTR----VTEE
C. elegans      213 NMRYMHYI-APEYEILDNTELTSAADIYSFGICSLEIAVIGGLSGCQNGSSE--GPVTED
D. melanogaster 282 RERGAHYFQAPEYGAADQ--LTAALDIYAFGMCALEMAALEIQP--SNSEST---AINEE
A. queenslandica 222 AAAGLHYA-APELAEGP---MRTSADIYAFGICALEMLNLALL---GNGETVQQGQLRSD

NRBP1           287 AISSAIQLLEDPLQREFIQKCLQSEPARRPTARELLFHPALFEVPSLKLLAAHCIV---G
NRBP2           266 AIARARHSLSDPNMREFILCCLARDPARRPSAHSLLFHRVLFEVHSLKLLAAHCFI---Q
C. elegans      270 VIEKAIRSLEDPMQQDFIRQCLRKDPAERPSARELLFHQILFEVHSLKLLSAHAIV---D
D. melanogaster 335 TIQRTIFSLENDLQRDLIRKCLNPQPQDRPSANDLLFHPLLFEVHSLKLLTAHCLVFSPA
A. queenslandica 275 AIQKALDQL-NPRTKQFIELCIDHDYEKRPQAHSLIKHLVLQEVFTLKLLSAYALR---G

NRBP1           344 HQHMIPENALEE-ITKNMDTSAVLAEIPAGPGREPVQTLYSQSPALE--LDKFLEDVRNG
NRBP2           323 HQYLMPENVVEE-KTKAMDLHAVLAELPR-PRRPPLQWRYSEVSFME--LDKFLEDVRNG
C. elegans      327 SKKY--EDVSES-AFRIKDNETIAATSKL------REMAYCQVAAFQVDLEKFLDDVRNG
D. melanogaster 395 NRTMFSETAFDGLMQRYYQPDVVMAQLRL-AGGQERQYRLADVSGAD-KLEKFVEDVKYG
A. queenslandica 331 VKNL--QDIIDS-YRKSSD--TVLATVNA----KRIQKTFTAADTAQIDIEKFFDDISQD

NRBP1           401 IYPLTAF-------------------------------------------------GLP-
NRBP2           379 IYPLMNFA--ATRP-----------------------------------------LGLP-
C. elegans      378 IYPLTAFAPLAHQPSTTLRAYSNTNPSTLITTDISAPSSTHPSANSTITAETSVNTSLPG
D. melanogaster 453 VYPLITYS--GKKP--------------------------------------------PN
A. queenslandica 382 LL----FE--AEQI-----------------------------------AEN--------

NRBP1           411 -RPQQP-----------------QQEEVTSPV--------------------------VP
NRBP2           395 -RVLAP-----------------PPEEVQ-------------------------------
C. elegans      438 -QSSQPSGTTTNTNGPSSIGKSASPEAVDKKIGEVTSTESTSKVEVEVNGANVTIGSSNG
D. melanogaster 467 FRSRAA-----------------SPERAD-------------------------------
A. queenslandica 393 -----------------------SPQEKDEPRRAGILTE---------------------

NRBP1           427 PSVKTPTPEP---------AEVETRKVVLMQCNIESVEEGVKHHLTLLLKLEDKLNRHLS
NRBP2           406 -KAKTPTPEP---------FDSETRKVIQMQCNLERSEDKARWHLTLLLVLEDRLHRQLT
C. elegans      497 RDAGSPTPEEEGEPNGERDMRLENRHILEINVHIENEE------MSIVLLLEDQMHRQLT
D. melanogaster 479 -SVKSATPEP---------VDTESRRIVNMMCSVKIKEDSNDITMTILLRMDDKMNRQLT
A. queenslandica 409 -SNSTATDRT------STNHELETRRFATCSCDVLSLEDG-KNEVCIKLQFGSKMERELR

NRBP1           478 CDLMPNENIPELAAELVQLGFISEADQSRLTSLLEETLN---------------------
NRBP2           456 YDLLPTDSAQDLASELVHYGFLHEDDRMKLAAFLESTFL---------------------
C. elegans      551 TSINKGDNPETLTENLITHGFMCQLDSEGVEKAIAVAFDIRAARIAEGVQEEENETSTRE
D. melanogaster 529 CQVNENDTAADLTSELVRLGFVHLDDQDKIQVLLEETLK--AGVMSDGAGAESSGAGVTT
A. queenslandica 461 IELQEDDTPQDLVADLVQWGLLSREDAEPLVSKLSTILA---------------------

NRBP1           517 ----------KFNFARNSTLNSA----------------------AVTVSS
NRBP2           495 ----------KYRGTQA----------------------------------
C. elegans      611 SNSEAPIE----NGTSSSITNSVKPIVDSVAPSSQTP--------TTTTSS
D. melanogaster 587 TATMAALEQLERNWSISSDADKQGTAVMYVPQEQQNADGDVDVEHSGTTSN
A. queenslandica    ---------------------------------------------------
```

Supplementary Fig. 11 Comparison of the protein sequences of NRBP1, NRBP2, and their homologous counterparts from selected invertebrate species. Conserved amino acids are highlighted in red, and similar residues are indicated in blue.

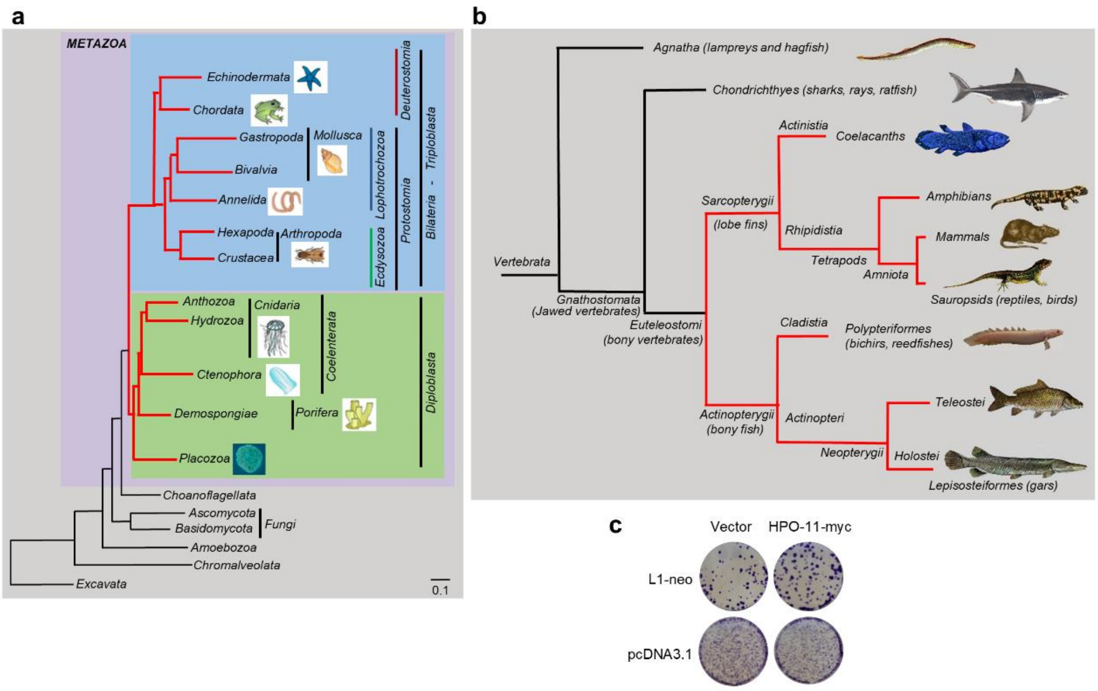

Supplementary Fig. 12 Metazoan origin of *NRBP* and the emergence of the paralog *NRBP2* in the Euteleostomi.

a, We mapped 2,065 homologs of human *NRBP1/2* to a phylogenetic tree of eukaryotes. Clades drawn with red lines indicate the presence of *NRBP*, whereas clades drawn with black lines indicate the absence of *NRBP*. *NRBP* is present in the early metazoan branches Porifera and Placozoa, but it is absent from all non-metazoan eukaryotes. The figure is a modified image from[10]. The original image is licensed under the Creative Commons Attribution 2.5 Generic license (https://creativecommons.org/licenses/by/2.5/deed.en).

b, We mapped 669 candidate orthologues of human *NRBP2* to a phylogenetic tree of vertebrates[11]. Clades drawn with red lines indicate the presence of *NRBP2*, whereas clades drawn with black lines indicate the absence of *NRBP2*. All *NRBP2* map either to the Sarcopterygii or to the Actinopterygii, indicating an evolutionary origin of *NRBP2* either in the Eutelestomi, or earlier in vertebrate evolution. All presented clades (black and red lines) have candidate orthologues of *NRBP1*. Remarkably, clades with

NRBP2 have a Type II L1 ORF1p, which is characterised by transposase 22, whereas the clades lacking *NRBP2* also lack the transposase 22. For details, please refer to the discussion section. The image is modified from https://en.wikipedia.org/wiki/Actinopterygii.

c, HPO-11 from *C. elegans* increases L1 activity. Shown is one representative picture of the colony assay depicted in the main **Fig. 7d**. n = 3 biological replicates with consistent results.

References:

1. Hwang, S.Y. *et al.* L1 retrotransposons exploit RNA m(6)A modification as an evolutionary driving force. *Nat Commun* **12**, 880 (2021).
2. Huntley, M.A. & Golding, G.B. Simple sequences are rare in the Protein Data Bank. *Proteins* **48**, 134-140 (2002).
3. Jumper, J. *et al.* Highly accurate protein structure prediction with AlphaFold. *Nature* **596**, 583-589 (2021).
4. Varadi, M. *et al.* AlphaFold Protein Structure Database in 2024: providing structure coverage for over 214 million protein sequences. *Nucleic Acids Res* **52**, D368-D375 (2024).
5. Wootton, J.C. & Federhen, S. Analysis of compositionally biased regions in sequence databases. *Methods Enzymol* **266**, 554-571 (1996).
6. Kerr, J.S. & Wilson, C.H. Nuclear receptor-binding protein 1: a novel tumour suppressor and pseudokinase. *Biochem Soc Trans* **41**, 1055-1060 (2013).
7. Hooper, J.D., Baker, E., Ogbourne, S.M., Sutherland, G.R. & Antalis, T.M. Cloning of the cDNA and localization of the gene encoding human NRBP, a ubiquitously expressed, multidomain putative adapter protein. *Genomics* **66**, 113-118 (2000).
8. Wang, X. *et al.* Interactions between HIV-1 Vif and human ElonginB-ElonginC are important for CBF-beta binding to Vif. *Retrovirology* **10**, 94 (2013).
9. Schieven, S.M. *et al.* The Elongin BC Complex Negatively Regulates AXL and Marks a Differentiated Phenotype in Melanoma. *Mol Cancer Res* **21**, 428-443 (2023).
10. Schierwater, B. *et al.* Concatenated analysis sheds light on early metazoan evolution and fuels a modern "urmetazoon" hypothesis. *PLoS Biol* **7**, e20 (2009).
11. Betancur, R.R. *et al.* The tree of life and a new classification of bony fishes. *PLoS Curr* **5** (2013).
