# Supplementary material for: Opposing roles of pseudokinases NRBP1 and NRBP2 in regulating L1 retrotransposition
Source: Nat Commun. 2025 Jul 11;16:6327. doi: 10.1038/s41467-025-61626-z (PMC12254500; doi:10.1038/s41467-025-61626-z)
Supplement: Supplementary file 2 — Description of Additional Supplementary Files [file 41467_2025_61626_MOESM2_ESM.pdf]

Description of Additional Supplementary Files

Supplementary Data 1: Analysis of NRBP1/2 interactors.

Supplementary Data 2: Analysis of RNA-seq data upon knockdown of NRBP1 or NRBP2.

Supplementary Data 3: Sequences, antibodies and key solutions.
