# Supplementary material for: Opposing roles of pseudokinases NRBP1 and NRBP2 in regulating L1 retrotransposition
Source: Nat Commun. 2025 Jul 11;16:6327. doi: 10.1038/s41467-025-61626-z (PMC12254500; doi:10.1038/s41467-025-61626-z)
Supplement: Supplementary file 6 — Reporting Summary [file 41467_2025_61626_MOESM6_ESM.pdf]

nature portfolio

Corresponding author(s): Wenjing Qi, Ralf Baumeister

Last updated by author(s): Jun 10, 2025

Reporting Summary

Nature Portfolio wishes to improve the reproducibility of the work that we publish. This form provides structure for consistency and transparency in reporting. For further information on Nature Portfolio policies, see our Editorial Policies and the Editorial Policy Checklist.

Statistics

For all statistical analyses, confirm that the following items are present in the figure legend, table legend, main text, or Methods section.

n/a	Confirmed	
☐	☒	The exact sample size (*n*) for each experimental group/condition, given as a discrete number and unit of measurement
☐	☒	A statement on whether measurements were taken from distinct samples or whether the same sample was measured repeatedly
☐	☒	The statistical test(s) used AND whether they are one- or two-sided<br>*Only common tests should be described solely by name; describe more complex techniques in the Methods section.*
☐	☒	A description of all covariates tested
☐	☒	A description of any assumptions or corrections, such as tests of normality and adjustment for multiple comparisons
☐	☒	A full description of the statistical parameters including central tendency (e.g. means) or other basic estimates (e.g. regression coefficient) AND variation (e.g. standard deviation) or associated estimates of uncertainty (e.g. confidence intervals)
☐	☒	For null hypothesis testing, the test statistic (e.g. *F*, *t*, *r*) with confidence intervals, effect sizes, degrees of freedom and *P* value noted<br>*Give P values as exact values whenever suitable.*
☒	☐	For Bayesian analysis, information on the choice of priors and Markov chain Monte Carlo settings
☒	☐	For hierarchical and complex designs, identification of the appropriate level for tests and full reporting of outcomes
☐	☒	Estimates of effect sizes (e.g. Cohen's *d*, Pearson's *r*), indicating how they were calculated

Our web collection on statistics for biologists contains articles on many of the points above.

Software and code

Policy information about availability of computer code

Data collection	Chemiluminescence signals were captured using the LAS-4000 imaging system (Fujifilm). Absorbance measurements were obtained at 590 nm using a microplate reader. qRT-PCR was conducted on the Light Cycler 96 System (Roche). Confocal fluorescence images were acquired using LSM-U-NLO or LSM-I-NLO confocal microscopes (Carl Zeiss), with Airyscan super-resolution mode when indicated.
Data analysis	Mass spectrometry (MS) data analysis:<br>Raw MS files were searched with MaxQuant version 2.4.9.0 against the homo sapiens Uniprot reference proteome (ID: UP000005640; 20594 protein entries; October 2022).<br>For MS data analysis, the proteingroups.txt file of Maxquant was used and loaded into Perseus 2.0.10.0.<br><br>RNA-seq data analysis was performed using the European Galaxy server (https://usegalaxy.eu/), and the tools used are listed below:<br>The quality of FASTQ files was checked by FastQC (v0.72).<br>Trimmed reads were aligned to the human genome USCS build hg38 using RNA STAR (v2.7.2b).<br>Cutadapt (v1.16.5) was used to remove the adapters.<br>The number of reads was counted by FeatureCounts (v1.6.4) using default parameters.<br>Differentially expressed genes were identified by EdgeR (v3.24.1).<br><br>Gene Ontology analysis: DAVID Bioinformatics Resources 6.8 (https://david.ncifcrf.gov/).<br>Statistical analysis and data visualization: Microsoft Excel 365 and GraphPad Prism 9.<br>Fluorescence intensity measurements and line profile analyses: ImageJ 1.53.

For manuscripts utilizing custom algorithms or software that are central to the research but not yet described in published literature, software must be made available to editors and reviewers. We strongly encourage code deposition in a community repository (e.g. GitHub). See the Nature Portfolio guidelines for submitting code & software for further information.

Data

Policy information about availability of data

All manuscripts must include a data availability statement. This statement should provide the following information, where applicable:

- Accession codes, unique identifiers, or web links for publicly available datasets
- A description of any restrictions on data availability
- For clinical datasets or third party data, please ensure that the statement adheres to our policy

The mass spectrometry proteomics data generated in this study have been deposited in the ProteomeXchange Consortium via the PRIDE partner repository under accession code PXD051452 (https://www.ebi.ac.uk/pride/archive/projects/PXD051452). The RNA-seq data have been deposited in BioProject under ID PRJNA1101872 (https://www.ncbi.nlm.nih.gov/bioproject/?term=PRJNA1101872). Uncropped Western blots are available in Source Data.
The association between NRBP2 expression and Rheumatoid Arthritis: Autoimmune Diseases Explorer (ADEX) platform (https://adex.genyo.es/).

Research involving human participants, their data, or biological material

Policy information about studies with human participants or human data. See also policy information about sex, gender (identity/presentation), and sexual orientation and race, ethnicity and racism.

Reporting on sex and gender	Not applicable. This study did not involve human participants. Only publicly available, de-identified data from the Autoimmune Diseases Explorer (ADEX) database (https://adex.genyo.es/) were used.
Reporting on race, ethnicity, or other socially relevant groupings	Not applicable.
Population characteristics	Not applicable.
Recruitment	Not applicable.
Ethics oversight	Not applicable.

Note that full information on the approval of the study protocol must also be provided in the manuscript.

Field-specific reporting

Please select the one below that is the best fit for your research. If you are not sure, read the appropriate sections before making your selection.

☒ Life sciences ☐ Behavioural & social sciences ☐ Ecological, evolutionary & environmental sciences

For a reference copy of the document with all sections, see nature.com/documents/nr-reporting-summary-flat.pdf

Life sciences study design

All studies must disclose on these points even when the disclosure is negative.

Sample size	Sample sizes were selected based on standards widely accepted in the field. Key references are Luqman-Fatah A. et al. (2023), Hwang S.Y. et al. (2021), and Tristán-Ramos P. et al. (2020), all published in Nature Communications.
Data exclusions	No data were excluded from the analyses unless there was clear technical failure, such as failed transfection.
Replication	Experiments were independently repeated two or more times. Replication details are provided in the figure legends.
Randomization	Samples were allocated randomly into experimental groups.
Blinding	Blinding was not applicable. The investigators were aware of the treatment conditions, as the experiments involved molecular or cellular assays. Quantitative analyses were performed using objective and consistent criteria.

Reporting for specific materials, systems and methods

We require information from authors about some types of materials, experimental systems and methods used in many studies. Here, indicate whether each material, system or method listed is relevant to your study. If you are not sure if a list item applies to your research, read the appropriate section before selecting a response.

Materials & experimental systems

n/a	Involved in the study
☐	☒ Antibodies
☐	☒ Eukaryotic cell lines
☐	☐ Palaeontology and archaeology
☐	☐ Animals and other organisms
☐	☐ Clinical data
☐	☐ Dual use research of concern
☐	☐ Plants

Methods

n/a	Involved in the study
☐	☐ ChIP-seq
☐	☐ Flow cytometry
☐	☐ MRI-based neuroimaging

Antibodies

Antibodies used

For Western blot:
Rabbit anti-ORF1p (1:1000 dilution, Abcam, ab230966, clone number: EPR22227-54)
Mouse anti-Flag (1:1000 dilution, Sigma, F1804, M2, RRID: AB_262044)
Mouse anti-G3BP1 (1:1000 dilution, Santa Cruz, sc-81940, TT-Y)
Rat anti-HA (1:1000 dilution, Roche, 11867423001, clone number: 3F10, RRID: AB_390918)
Rabbit anti-Myc (1:1000 dilution, Cell signalling, 2278, clone number: 71D10, RRID: AB_490778)
Rabbit anti-Flag (1:1000 dilution, Cell signalling, 14793, clone number: D6W5B, RRID: AB_2572291)
Rabbit anti-NRBP1/2 (1:1000 dilution, Proteintech, 21549-1-AP, RRID: AB_10733219)
Mouse anti-NRBP1 [6C11] (1:1000 dilution, GeneTex, GTX84003, clone number: 6C11)
Mouse anti-GAPDH (1:10000 dilution, Proteintech, 60004-1-Ig, clone number: 1E6D9, RRID: AB_2107436)
Rabbit anti-UPF1 (1:1000 dilution, Cell Signaling, 12040, clone number: D15G6)
Rabbit anti-MOV10 (1:1000 dilution, Abcam, ab80613),
Rabbit anti-YB1 (1:1000 dilution, Cell Signaling, 4202, clone number: D299, RRID: AB_1950384),
Rabbit anti-Elongin B (1:1000 dilution, Abcam, ab168836)
Rabbit anti-Elongin C (1:1000 dilution, Proteintech, 12450-1-AP, RRID:AB_2201139)

For Immunofluorescence:
Rabbit anti-ORF1p (1:25 dilution, Abcam, ab230966, clone number: EPR22227-54)
Mouse anti-DDX6 (1:100 dilution,Sigma, SAB4200837, clone number: DDX6-34)
Mouse anti-Flag (1:400 dilution, Sigma, F1804, M2, RRID: AB_262044)
Mouse anti-G3BP1 (1:200 dilution, Santa Cruz, sc-81940, TT-Y)
Rabbit anti-G3BP2 (1:200 dilution, Proteintech, 16276-1-AP, RRID: AB_2878237)
Rat anti-HA (1:200 dilution, Roche, 11867423001, clone number: 3F10, RRID: AB_390918)
Mouse anti-TIAR (1:50 dilution, Santa Cruz, sc-398372, clone number: G-6)
Rabbit anti-Myc (1:200 dilution, Cell signalling, 2278, clone number: 71D10, RRID: AB_490778)
Rabbit anti-Flag (1:400 dilution, Cell signalling, 14793, clone number: D6W5B, RRID: AB_2572291)

Validation

Rabbit anti-ORF1p (Abcam, ab230966): manufacturer validated for WB/IF; And validated by knockdown, see Extended Data Fig. 10a.
Mouse anti-Flag (Sigma, F1804): Widely used commercial antibody, manufacturer validated.
Mouse anti-G3BP1 (Santa Cruz, sc-81940): manufacturer validated; And validated by knockout, see Extended Data Fig. 3e.
Rat anti-HA (Roche, 11867423001): Manufacturer validated.
Rabbit anti-Myc (Cell Signaling, 2278): Manufacturer validated.
Rabbit anti-Flag (Cell Signaling, 14793): Manufacturer validated.
Rabbit anti-NRBP1/2 (Proteintech, 21549-1-AP): Dual recognition confirmed by shRNA knockdown of NRBP1 and NRBP2, see Fig. 3d.
Mouse anti-NRBP1 [6C11] (GeneTex, GTX84003): Manufacturer validated; Signal reduced upon NRBP1 knockdown, see Fig.2g.
Mouse anti-GAPDH (Proteintech, 60004-1-Ig): Manufacturer validated.
Rabbit anti-UPF1 (Cell Signaling, 12040): Manufacturer validated.
Rabbit anti-MOV10 (Abcam, ab80613): Manufacturer validated.
Rabbit anti-YB1 (Cell Signaling, 4202): Manufacturer validated.
Rabbit anti-Elongin B (Abcam, ab168836): Manufacturer validated; Signal reduced upon knockdown Extended Data Fig. 9d.
Rabbit anti-Elongin C (Proteintech, 12450-1-AP): Manufacturer validated; Signal reduced upon knockdown, see Extended Data Fig. 9d.
Mouse anti-DDX6 (Sigma, SAB4200837): Manufacturer validated; Specificity supported by literature (Senatore E. et al., EMBO Rep, 2025).
Rabbit anti-G3BP2 (Proteintech, 16276-1-AP): Manufacturer validated; Signal reduced upon knockdown, see Extended Data Fig. 3d.
Mouse anti-TIAR (Santa Cruz, sc-398372): Manufacturer validated.

Eukaryotic cell lines

Policy information about cell lines and Sex and Gender in Research

Cell line source(s)

HeLa alpha Kyoto cell line is a subclone of the original HeLa line established at Kyoto University. HEK293T cells (CRL-3216) are from ATCC. MCF-7 cells (ACC115) are provided by DSMZ (German Collection of Microorganisms and Cell Cultures).

Authentication

The cell lines were obtained from reputable sources and regularly monitored for morphology and growth characteristics.

Mycoplasma contamination	All cell lines were routinely tested and confirmed to be negative for mycoplasma contamination.
Commonly misidentified lines (See ICLAC register)	No.

Palaeontology and Archaeology

Specimen provenance	*Provide provenance information for specimens and describe permits that were obtained for the work (including the name of the issuing authority, the date of issue, and any identifying information). Permits should encompass collection and, where applicable, export.*
Specimen deposition	*Indicate where the specimens have been deposited to permit free access by other researchers.*
Dating methods	*If new dates are provided, describe how they were obtained (e.g. collection, storage, sample pretreatment and measurement), where they were obtained (i.e. lab name), the calibration program and the protocol for quality assurance OR state that no new dates are provided.*

☐ Tick this box to confirm that the raw and calibrated dates are available in the paper or in Supplementary Information.

Ethics oversight	*Identify the organization(s) that approved or provided guidance on the study protocol, OR state that no ethical approval or guidance was required and explain why not.*

Note that full information on the approval of the study protocol must also be provided in the manuscript.

Animals and other research organisms

Policy information about studies involving animals; ARRIVE guidelines recommended for reporting animal research, and Sex and Gender in Research

Laboratory animals	*For laboratory animals, report species, strain and age OR state that the study did not involve laboratory animals.*
Wild animals	*Provide details on animals observed in or captured in the field; report species and age where possible. Describe how animals were caught and transported and what happened to captive animals after the study (if killed, explain why and describe method; if released, say where and when) OR state that the study did not involve wild animals.*
Reporting on sex	*Indicate if findings apply to only one sex; describe whether sex was considered in study design, methods used for assigning sex. Provide data disaggregated for sex where this information has been collected in the source data as appropriate; provide overall numbers in this Reporting Summary. Please state if this information has not been collected. Report sex-based analyses where performed, justify reasons for lack of sex-based analysis.*
Field-collected samples	*For laboratory work with field-collected samples, describe all relevant parameters such as housing, maintenance, temperature, photoperiod and end-of-experiment protocol OR state that the study did not involve samples collected from the field.*
Ethics oversight	*Identify the organization(s) that approved or provided guidance on the study protocol, OR state that no ethical approval or guidance was required and explain why not.*

Note that full information on the approval of the study protocol must also be provided in the manuscript.

Clinical data

Policy information about clinical studies

All manuscripts should comply with the ICMJE guidelines for publication of clinical research and a completed CONSORT checklist must be included with all submissions.

Clinical trial registration	*Provide the trial registration number from ClinicalTrials.gov or an equivalent agency.*
Study protocol	*Note where the full trial protocol can be accessed OR if not available, explain why.*
Data collection	*Describe the settings and locales of data collection, noting the time periods of recruitment and data collection.*
Outcomes	*Describe how you pre-defined primary and secondary outcome measures and how you assessed these measures.*

Dual use research of concern

Policy information about dual use research of concern

Hazards

Could the accidental, deliberate or reckless misuse of agents or technologies generated in the work, or the application of information presented in the manuscript, pose a threat to:

No	Yes	
☐	☐	Public health
☐	☐	National security
☐	☐	Crops and/or livestock
☐	☐	Ecosystems
☐	☐	Any other significant area

Experiments of concern

Does the work involve any of these experiments of concern:

No	Yes	
☐	☐	Demonstrate how to render a vaccine ineffective
☐	☐	Confer resistance to therapeutically useful antibiotics or antiviral agents
☐	☐	Enhance the virulence of a pathogen or render a nonpathogen virulent
☐	☐	Increase transmissibility of a pathogen
☐	☐	Alter the host range of a pathogen
☐	☐	Enable evasion of diagnostic/detection modalities
☐	☐	Enable the weaponization of a biological agent or toxin
☐	☐	Any other potentially harmful combination of experiments and agents

Plants

Seed stocks	*Report on the source of all seed stocks or other plant material used. If applicable, state the seed stock centre and catalogue number. If plant specimens were collected from the field, describe the collection location, date and sampling procedures.*
Novel plant genotypes	*Describe the methods by which all novel plant genotypes were produced. This includes those generated by transgenic approaches, gene editing, chemical/radiation-based mutagenesis and hybridization. For transgenic lines, describe the transformation method, the number of independent lines analyzed and the generation upon which experiments were performed. For gene-edited lines, describe the editor used, the endogenous sequence targeted for editing, the targeting guide RNA sequence (if applicable) and how the editor was applied.*
Authentication	*Describe any authentication procedures for each seed stock used or novel genotype generated. Describe any experiments used to assess the effect of a mutation and, where applicable, how potential secondary effects (e.g. second site T-DNA insertions, mosiacism, off-target gene editing) were examined.*

ChIP-seq

Data deposition

☐ Confirm that both raw and final processed data have been deposited in a public database such as GEO.

☐ Confirm that you have deposited or provided access to graph files (e.g. BED files) for the called peaks.

Data access links<br>*May remain private before publication.*	*For "Initial submission" or "Revised version" documents, provide reviewer access links. For your "Final submission" document, provide a link to the deposited data.*
Files in database submission	*Provide a list of all files available in the database submission.*
Genome browser session<br>(e.g. UCSC)	*Provide a link to an anonymized genome browser session for "Initial submission" and "Revised version" documents only, to enable peer review. Write "no longer applicable" for "Final submission" documents.*

Methodology

Replicates	*Describe the experimental replicates, specifying number, type and replicate agreement.*
Sequencing depth	*Describe the sequencing depth for each experiment, providing the total number of reads, uniquely mapped reads, length of reads and whether they were paired- or single-end.*
Antibodies	*Describe the antibodies used for the ChIP-seq experiments; as applicable, provide supplier name, catalog number, clone name, and lot number.*

Peak calling parameters	*Specify the command line program and parameters used for read mapping and peak calling, including the ChIP, control and index files used.*
Data quality	*Describe the methods used to ensure data quality in full detail, including how many peaks are at FDR 5% and above 5-fold enrichment.*
Software	*Describe the software used to collect and analyze the ChIP-seq data. For custom code that has been deposited into a community repository, provide accession details.*

Flow Cytometry

Plots

Confirm that:

- [ ] The axis labels state the marker and fluorochrome used (e.g. CD4-FITC).
- [ ] The axis scales are clearly visible. Include numbers along axes only for bottom left plot of group (a 'group' is an analysis of identical markers).
- [ ] All plots are contour plots with outliers or pseudocolor plots.
- [ ] A numerical value for number of cells or percentage (with statistics) is provided.

Methodology

Sample preparation	*Describe the sample preparation, detailing the biological source of the cells and any tissue processing steps used.*
Instrument	*Identify the instrument used for data collection, specifying make and model number.*
Software	*Describe the software used to collect and analyze the flow cytometry data. For custom code that has been deposited into a community repository, provide accession details.*
Cell population abundance	*Describe the abundance of the relevant cell populations within post-sort fractions, providing details on the purity of the samples and how it was determined.*
Gating strategy	*Describe the gating strategy used for all relevant experiments, specifying the preliminary FSC/SSC gates of the starting cell population, indicating where boundaries between "positive" and "negative" staining cell populations are defined.*

- [ ] Tick this box to confirm that a figure exemplifying the gating strategy is provided in the Supplementary Information.

Magnetic resonance imaging

Experimental design

Design type	*Indicate task or resting state; event-related or block design.*
Design specifications	*Specify the number of blocks, trials or experimental units per session and/or subject, and specify the length of each trial or block (if trials are blocked) and interval between trials.*
Behavioral performance measures	*State number and/or type of variables recorded (e.g. correct button press, response time) and what statistics were used to establish that the subjects were performing the task as expected (e.g. mean, range, and/or standard deviation across subjects).*

Acquisition

Imaging type(s)	*Specify: functional, structural, diffusion, perfusion.*
Field strength	*Specify in Tesla*
Sequence & imaging parameters	*Specify the pulse sequence type (gradient echo, spin echo, etc.), imaging type (EPI, spiral, etc.), field of view, matrix size, slice thickness, orientation and TE/TR/flip angle.*
Area of acquisition	*State whether a whole brain scan was used OR define the area of acquisition, describing how the region was determined.*

Diffusion MRI ☐ Used ☐ Not used

Preprocessing

Preprocessing software	*Provide detail on software version and revision number and on specific parameters (model/functions, brain extraction, segmentation, smoothing kernel size, etc.).*

Normalization

If data were normalized/standardized, describe the approach(es): specify linear or non-linear and define image types used for transformation OR indicate that data were not normalized and explain rationale for lack of normalization.

Normalization template

Describe the template used for normalization/transformation, specifying subject space or group standardized space (e.g. original Talairach, MNI305, ICBM152) OR indicate that the data were not normalized.

Noise and artifact removal

Describe your procedure(s) for artifact and structured noise removal, specifying motion parameters, tissue signals and physiological signals (heart rate, respiration).

Volume censoring

Define your software and/or method and criteria for volume censoring, and state the extent of such censoring.

Statistical modeling & inference

Model type and settings

Specify type (mass univariate, multivariate, RSA, predictive, etc.) and describe essential details of the model at the first and second levels (e.g. fixed, random or mixed effects; drift or auto-correlation).

Effect(s) tested

Define precise effect in terms of the task or stimulus conditions instead of psychological concepts and indicate whether ANOVA or factorial designs were used.

Specify type of analysis: ☐ Whole brain ☐ ROI-based ☐ Both

Statistic type for inference

(See Eklund et al. 2016)

Specify voxel-wise or cluster-wise and report all relevant parameters for cluster-wise methods.

Correction

Describe the type of correction and how it is obtained for multiple comparisons (e.g. FWE, FDR, permutation or Monte Carlo).

Models & analysis

n/a	Involved in the study
☐	☐ Functional and/or effective connectivity
☐	☐ Graph analysis
☐	☐ Multivariate modeling or predictive analysis

Functional and/or effective connectivity

Report the measures of dependence used and the model details (e.g. Pearson correlation, partial correlation, mutual information).

Graph analysis

Report the dependent variable and connectivity measure, specifying weighted graph or binarized graph, subject- or group-level, and the global and/or node summaries used (e.g. clustering coefficient, efficiency, etc.).

Multivariate modeling and predictive analysis

Specify independent variables, features extraction and dimension reduction, model, training and evaluation metrics.
